# An Integrated Approach to Evaluate the Influence of Dietary *Olea europaea* L. Polyphenols on Physiological Stress, Intestinal Morphofunctional Traits, and Meat Quality in Neroametà Pigs: A Preliminary Study

**DOI:** 10.3390/ani16071009

**Published:** 2026-03-25

**Authors:** Maria Chiara Di Meo, Ilva Licaj, Vittorio Maria Mandrone, Chiara Attanasio, Paolo De Girolamo, Armando Zarrelli, Pasquale Vito, Romania Stilo, Ettore Varricchio

**Affiliations:** 1 Department of Science and Technology (DST), University of Sannio, 82100 Benevento, BN, Italy; mardimeo@unisannio.it (M.C.D.M.); ilva.licaj@unisannio.it (I.L.); vittorio.mandrone13@gmail.com (V.M.M.); vito@unisannio.it (P.V.); romstilo@unisannio.it (R.S.); 2 Department of Veterinary Medicine and Animal Production, University of Naples Federico II, 80137 Naples, NA, Italy; chiara.attanasio@unina.it (C.A.); degirola@unina.it (P.D.G.); 3 Department of Chemical Sciences, University of Naples Federico II, 80126 Naples, NA, Italy; zarrelli@unina.it

**Keywords:** olive, leaves, oleuropein, feed, antioxidants, animal welfare, lipids, morphology, intestine, tenderness, sustainability

## Abstract

The use of olive by-products in livestock farming is a valuable resource, given their high levels of bioactive compounds with antioxidant and health-promoting properties. This preliminary study adopted an integrated approach to evaluate the influence of dietary *Olea europaea* L. polyphenols on animal welfare, physiological stress response, intestinal morphofunctional traits, and meat quality in Neroametà finishing pigs, a novel *Casertana* × *Large White* genetic line (Neroametà). Thirty pigs reared under extensive farming conditions were randomly allocated to two groups (*n* = 15): a control group fed a standard diet (C) and a treatment group (OL) supplemented with 300 mg/head/day of olive polyphenolic extract for 90 days. The study focused on the systemic correlation between host health and product quality. Meat composition, rheological properties, meat antioxidant activity, stress parameters, and fatty acid profiles of the *longissimus lumborum* and *psoas major* muscles were analyzed. Results showed that the OL diet significantly modulated the HPA axis, as evidenced by a marked reduction in plasma ACTH and cortisol levels, alongside improved antioxidant status. These physiological changes were positively associated with a trophic effect on the intestinal mucosa, characterized by increased villus height and a more favorable villus/crypt ratio. Regarding meat quality, the OL group exhibited superior oxidative stability, optimized pH decline, and an improved intramuscular fatty acid profile (increased MUFA and n-3 PUFA, reduced SFA). Despite the pilot scale of 30 animals, these findings provide a solid foundation for characterizing the Neroametà breed. In conclusion, *Olea europaea* L. polyphenols act as a multi-level modulator, enhancing physiological resilience and meat quality, offering a sustainable strategy for high-quality pork production in line with circular economy and One Health principles.

## Simple Summary

By-products from the olive oil supply chain, such as olive (*Olea europaea* L.) leaves, are rich in bioactive compounds with antioxidant properties and can be sustainably used in animal feeding. This study evaluated the effects of dietary supplementation with olive polyphenols in finishing pigs from a novel *Casertana × Large White* crossbreed. For 90 days, 30 finishing pigs were assigned to two groups: one received a standard diet, and the other a diet supplemented with 300 mg/head/day of olive polyphenols. Results showed that supplemented pigs had improved growth performance, reduced stress behaviors, and lower plasma cortisol levels, indicating better animal welfare. Meat from the treated group exhibited improvements in rheological properties and fatty acid profile, characterized by higher n-3 and n-9 content, as well as a more favorable n-3/n-6 ratio. Antioxidant capacity in the muscle and intestinal morphology were also significantly enhanced. These findings support the use of olive polyphenol supplementation as a natural and effective strategy to improve production efficiency, meat quality, animal welfare, and sustainable pig farming in extensive systems.

## 1. Introduction

The valorization of agro-industrial by-products as alternative feed ingredients has gained increasing attention as a sustainable strategy to improve animal health, welfare, and product quality [1]. Among these, olive (*O. europaea* L.) by-products, particularly olive leaves, represent an abundant and renewable resource in Mediterranean countries, characterized by a high concentration of polyphenols with recognized antioxidant, anti-inflammatory, and antimicrobial properties [2,3]. Similar bioactive phenolic compounds have been isolated from other Mediterranean plants, confirming the relevance of low-molecular-weight phenols in antioxidant and antimicrobial defense mechanisms [4]. Olive leaves, in particular, are rich in polyphenols such as oleuropein, hydroxytyrosol, verbascoside, and luteolin-7-glucoside, which have been shown to improve the oxidative status, immune response, and metabolic efficiency in livestock species [5,6]. Phenolic esters and related aromatic derivatives isolated from other plant matrices have also demonstrated notable antioxidant activity, supporting their role in oxidative stress modulation [7]. Dietary inclusion of olive polyphenols has been associated with the endogenous antioxidant system, increasing superoxide dismutase (SOD), catalase (CAT), and glutathione peroxidase (GPx) activities while decreasing lipid peroxidation markers such as malondialdehyde (MDA) and improving meat oxidative stability in pigs, poultry, and ruminants [8,9]. In pigs, dietary supplementation with *Olea europaea* L. polyphenolic extract has been associated with improved oxidative status, greater muscle antioxidant capacity, and the enhanced shelf-life stability of meat [10]. These effects are primarily attributed to the strong radical scavenging capacity of oleuropein and its derivatives, which may also modulate the hypothalamic–pituitary–adrenal (HPA) axis, contributing to reduced physiological stress and improved welfare conditions [11]. In swine production, oxidative stress can negatively affect growth performance, carcass characteristics, and meat quality traits, including color, tenderness, and water-holding capacity [12]. Therefore, nutritional strategies aimed at enhancing antioxidant defense mechanisms are increasingly considered essential to improve both production efficiency and product quality [13]. Moreover, the inclusion of natural polyphenols in pig diets has been reported to influence the lipid composition of intramuscular fat, increasing the proportion of monounsaturated and polyunsaturated fatty acids (MUFA and PUFA), especially n-3 and n-9, while improving the n-6/n-3 ratio, key indicators of healthier and more functional meat [10,14].

Beyond their systemic antioxidant and metabolic effects, polyphenols may also exert protective actions at the intestinal level, contributing to gut integrity and nutrient absorption efficiency [15]. The intestinal mucosa plays a pivotal role in maintaining homeostasis, and its morphology, particularly villus height and crypt depth, is directly linked to absorptive capacity and barrier function [16]. Previous studies in pigs and poultry have shown that polyphenol-rich plant extracts can improve intestinal morphology, modulate gut microbiota composition, and increase villus height, thereby enhancing nutrient utilization and reducing inflammatory stress [17,18]. *Olea europaea* L. polyphenolic extract, in particular, have been shown to modulate intestinal architecture and tight junction integrity through antioxidant and anti-inflammatory mechanisms, thereby supporting a healthier gut environment [19].

The Neroametà pig, the focus of the present study, represents an emerging genetic line developed by crossing autochthonous Casertana boars with Italian Large White sows. This crossbreeding strategy is designed to synergize the high resilience and superior meat quality, specifically the favorable lipid profile, of the Casertana breed with the enhanced growth performance and lean meat yield characteristic of the Large White. Currently, this line is expanding within the Alto Tammaro production area (Province of Benevento, Italy), where it is reared under controlled semi-wild conditions in full compliance with biosafety regulations. The Neroametà exhibits remarkable adaptation to outdoor farming systems; indeed, the selection of this genetic line is driven by its superior productive performance and robustness compared to the purebred autochthonous Casertana. Given that the Neroametà pig is a non-consolidated genetic line, this introduction sets the stage for an exploratory investigation into its physiological responses. Specifically, this preliminary study aims to provide an initial experimental evaluation of the performance of the new Neroametà genetic line in response to a functional diet enriched with polyphenols, through an integrated analysis of its effects on growth performance, oxidative status, meat rheological properties, fatty acid composition, intestinal morphology, and stress-related hormonal profiles. The objective of this research was to conduct an experimental and empirical evaluation of the morpho-structural and qualitative parameters of the Neroametà breed, establishing a baseline for its future recognition and valorization. This study adopts an integrated approach to evaluate whether the antioxidant properties of *Olea europaea* L. polyphenols can mitigate physiological stress (as measured by ACTH and oxidative markers) and improve gut health, ultimately reflecting these systemic benefits on meat quality traits.

The primary objective of this preliminary study is to evaluate the effects of dietary supplementation with *Olea europaea* L. polyphenols not only on the morpho-structural parameters and animal welfare but also on the specific qualitative traits of the meat. By investigating two muscle groups (including *longissimus lumborum* and *psoas major*), this research aims to provide a preliminary characterization that supports the potential recognition and sustainable farming of the Neroametà pig. Furthermore, our findings are expected to provide new insights into the use of olive-derived bioactive molecules as natural and sustainable feed additives for enhancing both animal welfare and meat quality within pig production systems, particularly in extensive or semi-extensive rearing systems typical of Mediterranean areas.

## 2. Materials and Methods

All procedures described in this experiment were reviewed and approved by the Ethical Animal Care and Use Committee of the University of Naples “Federico II” (Protocol No. 175024-2025). Written informed consent was obtained from the owners for including their animals in this study.

### 2.1. Animals and Experimental Design

Thirty finishing male pigs Neroametà (*Casertana* boars × Italian *Large White* sow) reared at the “Mastrofrancesco” farm located in Morcone (Benevento, Italy) (41°20′01” N 14°41′59” E; 400 m above sea level) were used in this experiment. The newly developed pig genetic line, named “Neroametà”, was created by crossbreeding *Casertana* × *Large White*. This genetic line is not officially registered in any herdbook and was developed for experimental purposes. The sample size was chosen to establish a baseline for this non-consolidated genetic line, following the principles of the 3Rs and providing sufficient statistical power for an initial exploratory analysis of the integrated parameters.

Pigs were reared in an extensive system characterized by a controlled semi-wild management. Each dietary group was separated in large outdoor pens providing approximately 160–200 m^2^ per animal, ensuring a low stocking density that allowed for the expression of natural behaviors. The animals had constant outdoor access with grazing opportunities on spontaneous local vegetation, which supplemented their diet. Before the beginning of the trial, all piglets were reared under uniform conditions at the same facility, weaned at 60 days, and socialized in groups to ensure a homogeneous background. For the experiment, they were arranged in a completely randomized design. Male pigs were castrated after weaning and were divided into two dietary treatments (*n* = 15 per treatment), and all pigs were fed equal amounts of the same basal diet. To ensure biological replication and minimize pen effects, each group was further subdivided into three pens, each containing five animals, which served as the experimental units.

Animals were weighed (178 ± 7.00 kg initial body weight and 15 ± 1 month of age), individually identified, and fed once a day (3 kg/head/day) during the study period, while fresh water was provided ad libitum. The trial lasted 90 days. Residual feed was collected after each feeding to determine the average daily feed intake (DMI). Animals were weighed weekly to calculate the average daily gain (ADG) and feed conversion index (FCI). All animals were slaughtered on the same day at a commercial slaughterhouse, according to the European Union welfare guidelines (Council Regulation (EC) No. 1099/2009). Animals were electrically stunned and exsanguinated. The carcass weight was recorded 24 h after slaughter, and samples of the *longissimus lumborum (LL)*, sampling at the level of the first and second lumbar vertebrae, and *psoas major (PM)* muscles were taken from each carcass to assess meat quality and determine intramuscular fat content. Samples were transported to the laboratory under refrigerated conditions (4 °C) within 2 h of collection. Morphological evaluations were initiated immediately, while aliquots for analysis were stored at −80 °C until further processing.

### 2.2. Dietary Treatments

The dietary treatments were (i) the control group (C): standard diet based on barley, maize, soya, bran, and vitamin premix; (ii) the treated group (OL): standard diet +300 mg/head/day of *O. europaea* L. leaves powder extract. Dietary supplementation was based on the addition of a dry extract of olive leaves from the *Caiazzana* cultivar in powder form. The *Caiazzana* leaves were harvested in March, when they showed greater antioxidant activity and higher phenolic content [3]. The functional feed was administered to the pigs in the OL group at a concentration of 300 mg/head/day. In this study, the choice of the dose of extract to be administered was based on previous studies demonstrating the high antioxidant capacity of this plant matrix [3]. The basal diet was formulated to meet the requirements for all nutrients. Individual feedstuffs from each animal category were sampled weekly, and their composition was analyzed according to the AOAC method (AOAC,1995) [20]. The olive leaf extract was added “on top” to the standard diet. Therefore, the standard and OL diets were isoenergetic and isoproteic. The components and the chemical composition of the standard diets and the *Olea europaea* L. polyphenolic extract (OL diet) are given in Table 1. To ensure that the observed effects were attributable solely to the polyphenolic supplementation, the experimental diets were formulated to be isoenergetic and isolipidic, with comparable fatty acid profiles (Table 2). Particular care was taken to maintain consistent levels of saturated fatty acids (SFAs) and n-6 polyunsaturated fatty acids (PUFAs) across both the standard and OL treatments.

### 2.3. Characterization of Olea europaea L. Extract and Functional Diets

Standard diet, OL diet, and *Olea europaea* L. polyphenolic extract were obtained through microwave-assisted extraction (MAE), following the procedure described by Di Meo et al. [21]. The total polyphenols (TPC) and flavonoids (TFC) content, along with antioxidant activity, were determined using the methodology described by Di Meo et al. [3] and Di Meo et al. [10]. Additionally, phenolic compounds in the olive leaves were characterized using HPLC-UV according to Di Meo et al. [10].

#### 2.3.1. Phenolic Content and Antioxidant Activity

The *Olea europaea* L. polyphenolic extract used in this study is characterized by a high content of TPC, TFC, and significant radical scavenging activity (Inhibition %), as detailed in Table 3. This specific chemical profile was utilized to standardize the antioxidant dosage administered throughout the trial, ensuring the reproducibility of the dietary treatment.

#### 2.3.2. Phenolic Profile of *Olea europaea* L. Polyphenolic Extract

The phenolic profile of the *O. europaea* L. cv. *Caiazzana* polyphenol extract used in this study was characterized by HPLC-UV analysis, as shown in Figure S1. The chromatogram showed the main peaks corresponding to tyrosol (3), hydroxytyrosol (5), luteolin-7-O-glucoside (9), chlorogenic acid (10), quercetin (12), verbascoside (15), and oleuropein diglucoside (16) as the predominant compounds. Oleuropein diglucoside (0.125 ± 0.05 mg/g) and verbascoside (0.100 ± 0.05 mg/g) appeared to be the most abundant compounds. The concentrations of these phenolic compounds were determined by comparing retention times and absorption spectra with those of pure standards, as reported in Table S1. These findings agree with previous studies for Italian olive cultivars, particularly *Caiazzana* [3,22].

### 2.4. Growth Performance

Growth performance parameters of pigs were recorded throughout the experimental period (90 days). The body weight (BW) of pigs was individually recorded at the beginning, every 15 days during the experiment, and at the end of the experimental period using a cage equipped with a digital scale. Average daily gain (ADG), total dry matter intake (DMI), and feed conversion ratio (FCR; calculated as feed intake per unit of weight gain) were determined every week.

Feed intake (FI) was recorded per pen using a mobile feed trolley equipped with a calibrated scale, allowing for accurate quantification of the feed distributed to each pen. At each individual weighing session, residual feed was collected and weighed to determine actual feed consumption. FCR was calculated for each pen as the ratio between total feed intake and total weight gain, and expressed as the mean per pig (total pen feed intake/15 pigs per pen). These metrics provided a comprehensive evaluation of growth performance across dietary treatments.

### 2.5. Morphological Traits and Welfare Parameters

#### 2.5.1. Morphology and Morphometry of the Small Intestine

In pigs, following slaughter and exsanguination, the abdominal cavity was opened, and the entire gastrointestinal tract was carefully removed. For morphological analysis, three segments of approximately 2 cm each were collected from the duodenum (approximately 15 cm distal to the pylorus) and jejunum (at the midpoint of the small intestine). The sampling procedure followed the protocol described by Hedemann et al. [23]. Subsequently, tissue samples were processed and analyzed by scanning electron microscopy to assess intestinal morphology. A scanning electron microscope equipped with energy-dispersive X-ray spectroscopy (SEM-EDS) (Zeiss EVO 15 HD VPSEM, operating at 15 kV, coupled with an Xmax 80 EDS detector; Oxford Instruments (Abingdon, Oxfordshire, UK)) was used to examine representative intestinal samples. Specifically, a 0.5 cm^2^ portion was subsampled from each intestinal segment for imaging. Before imaging, specimens were gold-sputtered to ensure adequate surface conductivity. The procedure was performed according to Muller et al. [24] and Skrzypek et al. [25]. For morphometric evaluation, specifically to measure crypt depth and villus height, a digital microscope (Dino-Lite, AnMo Electronics Corporation, New Taipei City, Taiwan) with a magnification range of 20×–220×, integrated coaxial illumination, and flexible LED control (FLC) was employed. Ten well-oriented villi and ten crypts were measured for each intestinal segment to obtain a representative mean. The instrument was equipped with a 5.0-megapixel color CMOS sensor (Lumenera Corporation, Ottawa, Canada). Image acquisition was performed using DinoCapture 2.0 software, and morphometric analysis was carried out with ImageJ software, version 1.54p.

#### 2.5.2. Blood Sampling and Hormonal Stress Indicators

To avoid the confounding effects of chronic handling stress on the hypothalamic–pituitary–adrenal (HPA) axis, blood was collected once per animal at the time of slaughter. Sampling was standardized between 08:00 and 10:00 h to account for circadian cortisol fluctuations [26]. Each pig was gently restrained in a standing position to reduce handling stress, and blood was drawn from the jugular vein using sterile EDTA-coated vacuum tubes (10 mL). The samples were immediately placed on ice and centrifuged at 3000 × g for 15 min at 4 °C. Plasma was separated and stored at −80 °C until analysis [10]. Plasma ACTH and cortisol concentrations, markers of stress and metabolic status, were determined using a commercially available ELISA kit validated for porcine samples (FineTest, Wuhan, China). For each assay, 50 µL of plasma was used, and all samples were analyzed in duplicate following the manufacturer’s instructions.

### 2.6. Meat Quality Characterization

#### 2.6.1. Physical Analysis: pH and Color

The pH was measured on the LL and PM muscles at 24 h (pH_24_) post-mortem using a portable pH meter (Hanna Instruments, Woonsocket, RI, USA) equipped with a penetration electrode. The pH meters were calibrated using a pH 7.0 and pH 4.0 buffer. Meat color coordinates (CIE L*, a*, b*) were determined 24 h post-mortem on the freshly cut transverse section of the *longissimus lumborum* and *psoas major* muscles. Measurements were performed in triplicate at three different locations on the muscle surface after a 30 min blooming period at 4 °C, using a Minolta CR-400 colorimeter (Konica Minolta, Osaka, Japan) with a D65 illuminant and 10° observer, standardized against a white calibration plate. The average of the three readings was recorded for each sample.

#### 2.6.2. Chemical Composition Analysis of Meat

The chemical composition of the meat was assessed on a representative sample of 50 g of minced *longissimus lumborum (LL)* muscle. The analysis included the determination of moisture, ash, crude protein, and intramuscular fat levels, following the official AOAC procedures (AOAC, 2002) [27]. Before analysis, each muscle sample was carefully trimmed to remove subcutaneous fat and visible external connective tissue. The remaining muscle tissue, containing the intramuscular fat (IMF), was then homogenized using a professional grinder. Briefly, 2 g of the ground meat was placed into a pre-weighed crucible and dried at 100 °C overnight to determine the dry matter percentage (AOAC, method 950.46). The samples were then placed into a box furnace at 500 °C for 18 h to determine the ash percentage (AOAC, method 920.153). Crude protein was determined using the Kjeldahl procedure (AOAC, method 981.10) on 0.5 g of sample, and the protein percentage was calculated by multiplying the total nitrogen by 6.25. For the determination of total intramuscular fat (IMF) content, 5 g of muscle sample was analyzed following the Soxhlet method, ensuring that the fat dispersed within the muscle fibers was fully recovered and analyzed without prior physical separation. Subsequently, to determine the fatty acid profile, lipids were extracted from an additional 2.5 g of muscle using a chloroform–methanol (2:1 *v*/*v*) solution, according to the Folch method [28]. This cold extraction procedure was specifically chosen to preserve the integrity of the fatty acids and prevent thermal degradation during the lipid recovery process, ensuring an accurate characterization via gas chromatography.

#### 2.6.3. Physicochemical and Mechanical Properties: Cooking Loss and Tenderness

The physicochemical and mechanical properties were evaluated in duplicate, except for the Warner–Bratzler shear force (WBSF), which was performed on seven cores per muscle of each animal. Following 24 h post-mortem, the LL and PM muscles were excised, weighed, vacuum-packed, and stored at 4 °C for seven days. The water-holding capacity was assessed at 24, 48, and 96 h of storage. Each slice was lightly dried with paper and weighed to calculate the weight loss (purge) according to the procedure described by Honikel [29]. In relation to the cooking procedure, samples of LL and PM were placed in open bags and cooked in a water bath at 75 °C until a core temperature of 72 °C was reached. This procedure was conducted to determine the cooking loss and to prepare the samples for the WBSF analysis, which is the standard method for evaluating meat tenderness. The following day, after storing the samples overnight at 4 °C, WBSF measurements were performed using an Instron Universal 5565 testing machine (Instron Ltd., High Wycombe, UK) equipped with a Warner–Bratzler shearing device (G-R Manufacturing Company, Manhattan, KS, USA). Seven cores (2.54 cm diameter) were cut perpendicularly to the muscle fiber orientation. The applied parameters were load cell 50 kg, cross-head speed 250 mm/min.

#### 2.6.4. Fatty Acids Analysis of Meat

Two muscle types (*longissimus lumborum* and *psoas major*) were selected for analysis. For each pig, three samples of each muscle type were analyzed. The fatty acid profile was determined by gas chromatography (GC) as described in Di Meo et al. [10]. Briefly, lipids previously extracted via the Folch method were used to determine the lipid profile, including saturated fatty acids (SFAs), monounsaturated fatty acids (MUFAs), and polyunsaturated fatty acids (PUFAs). Specifically, 100 mg of the extracted lipids were used to prepare fatty acid methyl esters (FAMEs) [10]. The GC analysis was conducted using an Agilent 8890A GC system (Agilent Technologies, Santa Clara, CA, USA), equipped with a split/splitless injection port and a flame ionization detector (FID). An Agilent 7693A automatic liquid sampler was used for sample introduction. The analysis was performed on an Agilent HP-88 capillary column (100 m × 0.25 mm, with a film thickness of 0.20 µm).

#### 2.6.5. Antioxidant Activity and Lipid Peroxidation of Meat

##### Antioxidant Capacity

The meat samples (1 g), in accordance with Branciari et al. [30], were mixed with a solution of ethanol/HCL 0.1 M 9.9/0.1 (*v*/*v*) at pH 4.0, homogenized with an Ultra-Turrax homogenizer (Ultra-Turrax T25 Basic, IKA Labortechnik Janke & Kunkel GmbH, Stavfen, Germany) for 1 min, and then shaken for 2 min. The homogenates were centrifuged at 6000 rpm at 4 °C for 20 min, and the supernatant was used to evaluate the antioxidant capacity after filtration of the sample with a 0.22 μm syringe filter. The DPPH free radical scavenging activity of the ethanol extracts was measured using the method described by Blasi et al. [31] with some modifications. Briefly, a 0.06 mmol/L solution of DPPH in ethanol was prepared and left for 1 h in the dark at 4 °C; the 0.06 mM DPPH ethanol solution (3.9 mL) was added to the ethanol extracts (0.1 mL). The solutions were shaken and left for 30 min in the dark. The absorbance was measured at 517 nm with a UV–Vis spectrophotometer (Jasco 7850; JASCO Corporation, Easton, MD, USA) against a blank, and the DPPH radical scavenging activity was expressed as % inhibition by the sample according to the equation I% = [(Ac − As)/Ac] × 100, where Ac is the absorbance of the control reaction (containing all reagents except the tested compound) and As is the absorbance of the tested compound. All determinations were performed in triplicate.

##### Antioxidant Enzyme Activity

The endogenous antioxidant activity of catalase (CAT) and superoxide dismutase (SOD) was evaluated in aliquots of LL and PM muscles. The samples (5 g of muscle) were processed and analyzed according to the method described by Pardo et al. [32] using an Ultra-Turrax^®^ homogenizer (IKA-Werke GmbH & Co. KG, Staufen, Germany) and a microplate reader (Infinite F200 PRO Tecan Plate Readers, Männedorf, Switzerland) for detection. CAT activity was determined using a Catalase Assay Kit (Sigma-Aldrich, St. Louis, MO, USA) by monitoring the H_2_O_2_ decomposition as a consequence of the catalytic activity of CAT via spectrophotometric measurement at 240 nm. SOD was determined using the SOD Determination Kit (Sigma-Aldrich, St. Louis, MO, USA), generating superoxide radicals using a xanthine/xanthine oxidase system. The SOD activity, as an inhibition activity, was quantified by measuring the decrease in the color development at 440 nm.

##### Lipid Peroxidation of Meat

The determination of the oxidative stability of the muscle samples was carried out using the Lipid Peroxidation (MDA) Assay Kit (Sigma-Aldrich, St. Louis, MO, USA), as a single measurement on meat samples collected 24 h post-mortem, with spectrophotometric detection (Infinite F200 PRO Tecan Plate Readers, Männedorf, Switzerland) at a wavelength of 532 nm, according to Rey et al. [5]. After collection, samples were immediately vacuum-packaged and stored at −80 °C until analysis to preserve the basal oxidative status. This approach was chosen to evaluate the cumulative effect of the dietary treatment on muscle oxidative stability at the end of the experimental trial, rather than monitoring the progression of oxidation during refrigerated storage. Malondialdehyde (MDA) is a main product of secondary lipid oxidation. The results were expressed as nmol/mg of meat as an average value of three measurements per sample.

### 2.7. Statistical Analysis

Statistical analyses of data were performed using GraphPad Prism (GraphPad, San Diego, CA, USA), version 8.0 for Windows. Before analysis, the normality of data distribution was verified using the Shapiro–Wilk test, and the homogeneity of variances was assessed with Levene’s test. For parameters that did not follow a normal distribution, specifically ACTH and Cortisol, data were analyzed using the non-parametric Mann–Whitney U test. The chemical composition of pig meat and animal performance were analyzed using one-way ANOVA followed by the Newman–Keuls post hoc test to evaluate the effects of dietary treatments; while physicochemical and mechanical properties were analyzed using a two-way ANOVA, with diet and muscle as fixed factors, followed by Tukey’s post hoc test for multiple comparisons. Data related to antioxidant activity and fatty acid profiles were analyzed by two-way ANOVA, followed by Tukey’s post hoc test, considering diet (Control and OL) and muscle type (*longissimus lumborum* and *psoas major*) as fixed factors, including their interaction (diet × muscle). When the experimental design involved a single factor, one-way ANOVA followed by Tukey’s post hoc test was applied for multiple comparisons. For direct pairwise comparisons between two groups (hormonal parameters), an independent-sample *t*-test was used. Morphometric measurements of the small intestine were analyzed by one-way ANOVA, considering diet (Control vs. OL) as a fixed factor; each value represented the mean of measurements taken from at least ten villi and ten crypts per animal. Results are expressed as the mean ± SD, and differences were considered statistically significant at *p* < 0.05. The threshold of *p* < 0.001 was utilized to highlight highly significant differences, particularly for antioxidant activity and phenolic content assays.

## 3. Results

### 3.1. Growth Performance

Table 4 reports the results related to the growth performance of pigs in the C group and the OL group. Statistical analysis revealed no significant differences between dietary treatments for all analyzed parameters including initial and final body weight, carcass weight, dry matter intake (DMI), average daily gain (ADG), and feed conversion ratio (FCR) (*p* > 0.05).

### 3.2. Morphological Traits and Welfare Parameters

#### 3.2.1. Intestinal Morphology and Morphometry

Morphological and morphometric analyses of the small intestine revealed an intact mucosal architecture with regularly shaped villi and crypts in both experimental groups.

**Figure 1:**
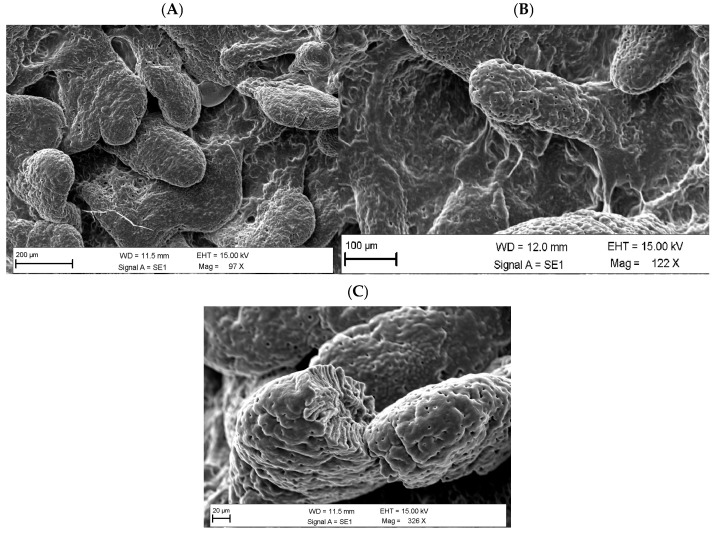
Scanning electron microscopy (SEM) image of the small intestinal villus from pigs fed the control diet (**A**) (97×, scale bar = 200 μm) and the OL diet (**B**) (122×, scale bar = 100 μm). SEM micrograph showing a cross-section of an intestinal villus from a pig receiving *Olea europaea* L. polyphenolic extract supplementation (326×, scale bar = 20 μm) (**C**). Regular, finger-like villi and intact epithelial surfaces are visible.

Scanning electron microscopy (SEM) revealed specific ultrastructural features: in the OL group, villi appeared more compact and organized compared to the control group, with a smoother and more continuous epithelial surface (Figure 1). Specifically, in Figure 1A, the mucosal surface was characterized by less-defined cellular elements and greater surface irregularity. In Figure 1B, the group treated with *O. europaea* extract showed a more uniform distribution of structures and an increase in villus height. In Figure 1C, the sectioned villus shows well-preserved tissue integrity and an active epithelial surface, likely reflecting high cellular turnover and efficient absorptive capacity. The sectioned villus shows defined folds and deep grooves.

The morphological findings align with the reported morphometric parameters (Table 5), demonstrating that dietary supplementation with *Olea europaea* L. polyphenolic extract resulted in a statistically significant increase in both villus height and crypt depth, further supporting the observed improvements in tissue structure and function. As shown in Table 5, dietary supplementation with *O. europaea* L. polyphenolic extract resulted in a statistically significant increase in both villus height and crypt depth compared to the control group (*p* < 0.05).

#### 3.2.2. Blood Stress Indicators

As shown in Table 6, plasma cortisol and ACTH concentrations were significantly reduced in pigs fed the OL diet compared to the control group. Specifically, cortisol levels showed a significant reduction from 9.52 ± 0.22 ng/mL in the control group to 6.07 ± 0.5 ng/mL in the OL group (*p* < 0.05), while ACTH levels decreased from 40.10 ± 3.0 pg/mL to 25.01 ± 2.10 pg/mL (*p* < 0.05), indicating that the OL diet effectively lowered these hormonal markers of stress. These results suggest that supplementation with the *Caiazzana* polyphenolic extract may modulate the hypothalamic–pituitary–adrenal axis, thereby reducing physiological stress in finishing pigs and potentially enhancing animal welfare under extensive rearing conditions.

### 3.3. Meat Quality Characterization

#### 3.3.1. Physical Traits of Meat: pH and Color

The dietary supplementation with *Olea europaea* L. extract significantly affected the physical traits of the meat. As shown in Table 7, the OL group exhibited significantly higher pH_24_ values in both the LL and PM muscles compared to the CTR group (*p* < 0.01). This might suggest that the antioxidant compounds in the extract may modulate the post-mortem glycolytic rate, preventing an excessive pH drop. Regarding color parameters, a significant effect of the diet was observed for the redness index (a*), which was higher in the OL group for both muscles (*p* < 0.05). This improvement in red color intensity could be attributed to the protective effect of olive polyphenols against myoglobin oxidation. The two-way ANOVA also confirmed a significant effect of the muscle type (*p* < 0.001); as expected, the *psoas major* (oxidative muscle) showed lower lightness (L*) and higher redness (a*) values compared to the *longissimus lumborum* (glycolytic muscle). No significant interaction (Diet × Muscle) was found, indicating that the polyphenolic extract consistently exerts a beneficial effect on meat color stability, regardless of the muscle’s specific metabolic profile.

#### 3.3.2. Meat Chemical Composition

Table 8 reports the results related to the chemical composition of pig meat in the C and OL groups. No significant differences were found in the chemical composition of the meat between the two groups (C and OL) (*p* > 0.05).

#### 3.3.3. Physicochemical and Mechanical Properties of Meat

The physicochemical and mechanical properties of pig meat are indicators of its textural quality and consumer acceptability. Parameters such as cooking loss (a physicochemical trait) and initial force, WBSF, peak force, and work (mechanical traits) were measured to evaluate the technological and structural integrity of the muscle tissue. Our results showed that the dietary treatment influenced the textural behavior of the meat, with samples from pigs receiving the *Olea europaea* L. polyphenolic extract supplementation exhibiting significantly higher initial force, peak force, and work compared to the control group. The Warner–Bratzler shear force (WBSF) measurements, which reflect the tenderness of the meat, were in line with the observed mechanical parameters: the PM muscle showed higher WBSF than the LL muscle, indicating a naturally firmer texture, while the OL diet increased WBSF in both muscles, suggesting enhanced structural resistance to shear. Table 9 presents the physicochemical and mechanical properties of the LL and PM muscles, highlighting significant effects of both dietary treatment and muscle type. In particular, pigs receiving the OL diet showed significantly reduced cooking loss (*p* < 0.001), and higher values of initial force, peak force, and total work (*p* < 0.05). These data might indicate a potential effect on post-mortem glycolysis, an improvement in water-holding capacity and structural consistency of the meat, suggesting increased mechanical resistance and enhanced technological quality of the product. Although a different trend might be expected based on the existing literature, our results showed this specific pattern. This discrepancy could be attributed to the specific genotype–environment–diet interaction, as autochthonous breeds like the Neroametà may possess distinct muscle fiber characteristics or metabolic rates that influence post-mortem traits differently when supplemented with polyphenols. Moreover, significant differences were observed between the two muscles: the PM muscle exhibited higher initial force, WBSF, peak force, and work values compared to the LL muscle, regardless of diet (*p* < 0.001). In summary, administration of the OL diet improved the mechanical properties of pork by increasing structural stability, water retention, and firmness, with differentiated effects between muscles. These results could be consistent with a potential increase in muscle structure stability and suggest a possible improvement in meat quality, potentially linked to bioactive compounds present in the OL diet.

#### 3.3.4. Fatty Acid Profile of Meat

The fatty acid composition of the *longissimus lumborum* and *psoas major* muscles, as affected by the dietary treatment and muscle type, is reported in Table 10. Dietary supplementation with *Olea europaea* L. extract significantly modified the lipid profile of the meat. Regarding saturated fatty acids (SFAs), the OL group showed a significant reduction in total SFA compared to the Control group (34.00% vs. 38.87%; *p* < 0.001), primarily driven by lower levels of palmitic (*p* < 0.001) and stearic (*p* < 0.001) acids. Conversely, the OL diet led to a significant increase in total monounsaturated fatty acids (MUFAs) (55.00% vs. 51.14%; *p* = 0.030), with higher concentrations of oleic acid (*p* = 0.025) and nervonic acid (*p* < 0.001). Total polyunsaturated fatty acids (PUFAs) were also significantly higher in the OL group (11.51% vs. 10.20%; *p* < 0.001). Within the PUFA class, the olive extract supplementation resulted in a substantial enrichment of n-3 fatty acids (2.04% vs. 1.08%; *p* < 0.001), specifically alpha-linolenic (ALA) and eicosapentaenoic (EPA) acids (*p* < 0.001). As a consequence of these changes, the n-6/n-3 ratio was significantly improved in the OL group compared to the Control (4.63 vs. 8.43; *p* < 0.001). Regarding the muscle effect, no significant differences were observed between the LL and PM for the main fatty acid classes or individual fatty acids (*p* > 0.05). Furthermore, no significant Diet × Muscle (D × M) interaction was found, indicating that the polyphenolic extract consistently influenced the fatty acid deposition regardless of the specific muscle metabolic profile.

#### 3.3.5. Antioxidant Activity and Lipid Peroxidation of Meat

Table 11 highlights significant differences in antioxidant enzyme activities and oxidative stress markers in both the LL and PM muscles of pigs fed the control diet and those receiving the OL diet supplemented with *O. europaea* polyphenols. Total antioxidant activity (TAA), superoxide dismutase (SOD), and catalase (CAT) activities were markedly higher in the OL-fed group compared with the control in both LL and PM muscles of the OL group (*p* < 0.001), indicating an enhanced antioxidant defense capacity. Conversely, malondialdehyde (MDA) levels, an indicator of lipid peroxidation, were significantly reduced in OL-fed pigs (*p* < 0.001), suggesting lower oxidative damage. A comparison between muscles revealed that PM generally exhibited higher antioxidant enzyme activities and lower MDA concentrations than LL under both dietary conditions (*p* < 0.001). These findings demonstrate that olive leaf supplementation effectively improves the antioxidant defense system, the redox balance, and reduces oxidative stress in pig muscle tissues, with consistent effects observed across different muscle types. Similar trends have been reported for other plant-derived metabolites, highlighting the multifunctional nature of phytochemicals in animal and environmental systems [33].

## 4. Discussion

The results of this preliminary study suggest that dietary supplementation with *Olea europaea* L. polyphenolic extract exerts a multi-level beneficial effect on Neroametà pigs, integrating physiological welfare, intestinal health, and meat quality. This systemic perspective is particularly relevant for the Neroametà pig, a non-consolidated genetic line. In this breed, the interaction between genotype, metabolism, and diet may strongly influence both productive and qualitative traits. Our findings suggest that a better morphofunctional state of the intestine supports a more balanced physiological response to stress, which in turn is reflected in both the meat’s pH and oxidative stability, highlighting the interconnection between gut health, stress physiology, and post-mortem muscle metabolism.

The increase in antioxidant activity observed in the OL diet compared to the standard diet suggests that the enrichment had a functional rather than merely analytical impact. This indicates not only a higher phenolic concentration but also an enhanced ability of the overall dietary matrix to neutralize free radicals and support oxidative balance, consistent with evidence that polyphenol-enriched feeds exert synergistic antioxidant effects beyond their individual compounds [34,35,36]. The enhanced antioxidant status may also interact with pH stabilization in the muscle, reducing protein denaturation and oxidative damage, which is particularly important when considering differences between muscles with distinct fiber types.

The absence of significant differences in growth performance parameters between pigs fed the Control and the OL diet suggests that dietary supplementation with *O. europaea* polyphenols did not adversely affect feed intake, nutrient utilization, or growth efficiency. Both groups exhibited comparable values for body weight, average daily gain, dry matter intake, and feed conversion ratio, indicating that the inclusion of olive leaf extract was well-tolerated and did not impair productive performance. These findings agree with previous studies showing that supplementation with olive leaves or phenolic-rich extracts does not significantly modify growth performance in pigs or other monogastric species [37,38]. Paiva-Martins et al. [37] demonstrated that supplementing pig diets with up to 1% olive leaf extract did not affect feed intake, average daily gain, or feed conversion ratio, despite a measurable increase in plasma antioxidant capacity. Similarly, Botsoglou et al. [38] observed that the inclusion of olive leaves in pig diets did not impair growth performance, supporting the notion that phenolic-rich feed ingredients can be used without negatively affecting productive parameters.

The lack of differences in carcass weight and *longissimus lumborum* chemical composition further supports the nutritional safety of the OL-supplemented diet. Similarly, the chemical parameters of the psoas major muscle remained stable across treatments, indicating that the inclusion of olive polyphenols does not adversely affect the main proximate constituents of different muscle types. This consistency between LL and PM muscles suggests that the dietary intervention maintains the core nutritional quality of the meat while specifically targeting the lipidomic and oxidative profile [39]. As observed by Hoz et al. [39], dietary modifications in pigs can significantly affect the fatty acid profile of the psoas major without altering its proximate composition. Similar results have been observed by Paié Ribeiro et al. [40], who reported that inclusion of up to 10% olive pomace in Bísaro pig diets did not compromise meat quality, with several parameters remaining stable (ash, total fat, and SFA) despite some variations in other components. These results support the idea that dietary olive by-products can be included in pig diets without negatively affecting muscle proximate composition, while potentially enhancing oxidative stability and extending meat shelf-life. Although no compositional changes were detected in the present study, the antioxidant compounds supplied through the OL diet may exert protective effects at the cellular level, particularly under oxidative or metabolic stress [41].

The rheological properties of pork are key indicators of meat quality, reflecting muscle structure, water-holding capacity, and texture [42]. In this study, pigs receiving the OL diet exhibited higher pH, reduced cooking loss, and increased initial force, peak force, and total work in both the LL and PM muscles compared to controls. The observed increase in pH is central to interpreting these results: pre-slaughter stress accelerates glycogen depletion, increasing post-mortem glycolysis and lactic acid accumulation, which lowers meat pH [43]. In contrast, OL-fed pigs showed reduced stress, likely preserving glycogen reserves and slowing post-mortem acidification. This mechanism links intestinal health, stress physiology, and meat quality, and explains improvements in tenderness, water-holding capacity, and mechanical resistance. The higher pH also interacts with antioxidant effects to stabilize myofibrillar proteins, further enhancing meat texture [43,44].

Muscle-specific differences were evident: the psoas major, rich in oxidative fibers, naturally showed higher initial force and work than the *longissimus lumborum*, a pattern reinforced by the OL diet [45]. These differences, attributable to fiber composition and intrinsic muscle structure, influence responses to cooking, shear, and oxidative stress [8]. The integration of pH, antioxidant capacity, and muscle type explains why the OL diet improved both the LL and PM properties, with a more pronounced effect in PM due to its oxidative profile. Similar positive effects on meat quality have been observed in studies using diets enriched with antioxidants or functional lipid precursors [46,47].

The improvement in meat color, particularly the increase in redness (a*), can also be interpreted in light of the enhanced antioxidant status. Polyphenols are known to protect myoglobin from oxidation, thereby stabilizing the red color of meat [44]. WBSF values for Neroametà (35.4 and 32.1 N for LL and PM, respectively) align with other Italian local breeds, confirming that olive polyphenols preserve the excellent meat quality traits typical of autochthonous Mediterranean lines [48,49].

Overall, these results demonstrate that olive leaf supplementation improves the technological quality of pork by enhancing texture, water-holding capacity, and structural stability without negatively affecting growth performance. The observed changes in rheological properties can therefore be interpreted as a downstream effect of integrated modulation of stress physiology, antioxidant defenses, and post-mortem metabolism [34,50].

In addition, dietary polyphenols significantly enhanced the antioxidant defense system in both muscles. Total antioxidant activity, superoxide dismutase, and catalase activities were significantly higher in OL-fed pigs, while malondialdehyde levels were reduced, indicating lower lipid peroxidation and improved oxidative stability. These findings support the mechanistic link between oxidative protection and improved meat quality, showing that antioxidant defenses help preserve pH-dependent protein structure and texture [6,51,52]. Muscle-specific differences were evident, with PM showing higher enzyme activities and lower MDA levels than LL, consistent with its oxidative fiber composition.

Dietary supplementation with *O. europaea* polyphenols significantly modulated the intramuscular fatty acid profile in both LL and PM muscles. Pigs receiving the OL diet exhibited reduced total SFA, primarily due to lower stearic acid, and increased MUFA and n-3 PUFA, including oleic acid, alpha-linolenic acid, and eicosapentaenoic acid. The improved n-6/n-3 ratio (4.63) reflects the nutritional relevance of the OL diet and suggests interactions between muscle fiber type, lipid metabolism, and dietary antioxidants, with oxidative fibers in PM preferentially incorporating unsaturated fatty acids [10,40,53,54,55].

A key finding concerns physiological stress. The significant reduction in plasma cortisol and ACTH concentrations in OL-fed pigs indicates modulation of the hypothalamic–pituitary–adrenal axis. Reduced stress likely contributed to both the higher meat pH and the improved antioxidant status, linking welfare and meat quality [5,56].

Finally, ultrastructural analyses revealed significant improvements in intestinal architecture, including increased villus height and crypt depth. These morphological adaptations enhance nutrient absorption and gut barrier function, creating a positive feedback loop: a healthier gut ensures more effective absorption of dietary polyphenols, which reach muscle tissues and exert antioxidant effects post-mortem [43,57,58,59,60,61,62].

Taken together, these results demonstrate that olive leaf polyphenols act as multifunctional modulators, linking intestinal health, oxidative balance, stress physiology, and muscle metabolism. Improvements in meat quality traits are thus the result of an integrated biological response, in which pH modulation, antioxidant protection, and muscle fiber characteristics play central roles [34,50].

Future studies including longer feeding periods, additional muscles, and broader physiological indicators will help validate these findings. Incorporating histochemical, transcriptomic, and sensory analyses will further clarify the molecular mechanisms and functional relevance of olive polyphenols in sustainable pig production.

## 5. Conclusions

In conclusion, this study provides the first comprehensive characterization of the Neroametà pig line, fulfilling our objective to define its specific physiological and qualitative traits. The results demonstrate that this previously unrecognized line is highly responsive to natural antioxidant supplementation, which significantly enhances meat stability and animal welfare. Specifically, the OL diet was associated with improvements in meat technological properties, modulation of intramuscular fatty acid profiles by increasing MUFA and n-3 PUFA while reducing SFA and the n-6/n-3 ratio, enhanced antioxidant defenses, lower oxidative stress, decreased plasma cortisol and ACTH levels, and a mild trophic effect on intestinal morphology.

These findings should be considered preliminary, as the study was limited to a pilot group of 30 animals and focused on only two muscles. Nevertheless, the integrated data provide a foundation for further research on the Neroametà pig line. Future studies including larger cohorts, additional muscles, longer feeding periods, and broader physiological and performance indicators will be necessary to confirm these observations.

Overall, these results highlight the potential of olive leaf polyphenols as a natural and sustainable feed additive for producing high-quality pork while valorizing agro-industrial by-products. In Mediterranean regions with high olive cultivation, this strategy could offer a valuable approach to enhance the functional value of meat, reduce the environmental impact of olive oil by-products, and promote their use as a local feed resource, in line with circular economy principles and the One Health concept. Within this context, dietary polyphenols may support better physiological resilience and adaptation for Neroametà pigs in sustainable farming, although further studies are required to validate these potential benefits.

## Figures and Tables

**Table 1:** Components and chemical composition of the standard diet and OL diet on dry matter (% D.M.).

	Standard Diet	OL Diet
Components	% D.M.	% D.M.
Maize	25.00	25.00
Barley	50.00	50.00
Soy protein 46%	10.00	10.00
Bran	12.50	12.50
Vitamin and mineral premix *	2.50	2.50
Parameters	% D.M.	% D.M.
Moisture	11.30	10.90
Dry matter	88.70	89.10
Crude protein	15.28	15.28
Ash	4.97	4.53
Crude fats	3.30	3.45
Crude fiber	4.08	4.15
Starch	44.56	44.56

* The premix contained the following quantities of vitamins, amino acids, and minerals: 300,000 IU/kg vitamin A, 2850 IU/kg vitamin D3, 1000 mg/kg vitamin E, 70 mg/kg vitamin K3, 100 mg/kg vitamin B1, 200 mg/kg vitamin B2, 100 mg/kg vitamin B6, 350 mg/kg calcium-D-pantothenate, 2 mg/kg vitamin B12, 900/kg mg niacin, 12,000 mg/kg choline chloride, 24 mg/kg folic acid, 3.4 mg/kg biotin, 4250 mg betaine, 7200 mg/kg Fe, 875 mg/kg Cu, 2050 mg/kg Mn, 6000 mg/kg Zn, 90 mg/kg I, 10 mg/kg Se, 1.98% lysine, 0.01% methionine, 23% calcium, 1.20% phosphorus, 7.30% sodium. Both diets were formulated to be isoenergetic and isoproteic.

**Table 2:** Fatty acid composition of the standard and OL pig diets (g/100 g fatty acids) ^a^.

Fatty Acid	Items	Standard Diet (g/100 g)	OL Diet (g/100 g)
Myristic	C14:0	0.88	0.70
Palmitic	C16:0	38.44	39.01
*Trans*-Palmitoleic	C16:1n 7t	0.09	0.05
Palmitoleic	C16:1n 7	0.15	0.18
Stearic	C18:0	10.73	11.01
Oleic	C18:1 n9	16.35	17.25
*Trans*-Linoleic	C18:2 n6t	0.30	0.18
Linoleic	C18:2 n6	30.96	29.11
Gamma-Linoleic	C18:3 n6	0.16	0.12
Eicosenoic	C20:1 n9	0.10	0.08
Alpha-Linolenic	C18:3 n3	0.08	1.01
Eicosadienoic	C20:2 n6	0.19	0.08
Dihomo-γ-Linolenic	C20:3 n6	0.12	0.10
Arachidonic	C20:4 n6	0.77	0.68
Lignoceric	C24:0	0.15	0.14
Eicosapentaenoic (EPA)	C20:5 n3	0.23	0.05
Nervonic	C24:1 n9	0.06	0.05
Docosahexaenoic (DHA)	C22:6 n3	0.01	0.01
Total			
	SFA	50.20	50.86
	MUFA	16.84	17.67
	PUFA	32.96	31.47

^a^ The control group (C) received feed (standard diet) without supplementation, and the experimental groups (OL) were offered the same diet supplemented with 300 mg/head/day of olive leaf polyphenol extract.

**Table 3:** Total phenolic content and antioxidant activity in standard diet (C), OL diet, and *Caiazzana* olive leaf ^1^.

Phenolic Extracts	TPC (mg GAE/g)	TFC (mg QE/g)	Inhibition %
Standard diet	9.05 ± 0.29	1.54 ± 0.23	25.01 ± 1.11
*Caiazzana* olive extract	22.20 ± 0.60	3.65 ± 0.25	50.40 ± 2.61
OL diet	36.09 ± 1.06	4.68 ± 0.32	66.10 ± 3.01

^1^ Abbreviation. TPC: Total Phenolic Content; TFC: Total Flavonoid Content; GAE: Gallic Acid Equivalents; QE: Quercetin Equivalents. Data are expressed as mean ± SD (*n* = 3). One-way ANOVA analysis between TPC, TFC, and inhibition% in phenolic extracts. TPC and TFC vs. Inhibition% in the standard diet, *Caiazzana* olive extract, OL diet (*p* < 0.001).

**Table 4:** Growth performances of Neroametà pigs ^1^.

	Dietary Treatment		*p*-Value
	Control Group	OL Group	
Initial BW, kg	178 ± 5.41	176 ± 5.63	0.189
Final BW, kg	200 ± 5.01	195 ± 5.30	0.256
Carcass weight, kg	170 ± 3.24	165 ± 2.91	0.539
DMI, g/d	2150 ± 13.61	2160 ± 13.58	0.104
ADG, g/d	244 ± 8.32	211 ± 8.59	0.633
FCR	8.81 ± 1.51	10.24 ± 1.03	0.316

^1^ Abbreviations: BW, body weight; DMI, dry matter intake; ADG, average daily gain; FCR, feed conversion ratio.

**Table 5:** Morphometric measurements of intestinal villus height and crypt depth in finishing pigs fed the control or olive leaf (OL) diet ^1^.

Parameter	Control Diet	OL Diet	*p*-Value
Villus height (μm)	395 ± 0.15	400 ± 0.17	*p* < 0.05
Crypt depth (μm)	140 ± 0.10	141 ± 0.10	*p* < 0.05

^1^ Values are mean ± SD (*n* = 15). Differences between groups (Control Diet vs. OL Diet) were assessed using a parametric *t*-test; *p* < 0.05.

**Table 6:** Plasma concentrations of ACTH (pg/mL) and cortisol (ng/mL) levels in finishing pigs ^1^.

Hormonal Parameter	Control Diet	OL Diet	*p*-Value
Cortisol (ng/mL)	9.52 ± 0.22	6.07 ± 0.50	*p* < 0.05
ACTH (pg/mL)	40.10 ± 3.0	25.01 ± 2.10	*p* < 0.05

^1^ Values are mean ± SD (*n* = 15). For non-normally distributed parameters (ACTH and Cortisol), significant differences between groups (Control Diet vs. OL Diet) were determined using the non-parametric Mann–Whitney U test.

**Table 7:** pH and color measurement of the *longissimus lumborum (LL)* and *psoas major* (PM) muscles in pigs ^1^.

Items	Diet (D)		Muscle (M)		*p*-Value		
	Control (Mean ± SD)	OL (Mean ± SD)	LL (Mean ± SD)	PM (Mean ± SD)	D	M	D × M
pH 24 h	5.61 ± 0.05	5.63 ± 0.04	5.58 ± 0.03	5.66 ± 0.06	<0.01	<0.001	0.125
L* (Lightness)	48.52 ± 2.1	47.90 ± 1.8	49.20 ± 2.0	47.22 ± 1.5	0.110	0.025	0.450
a* (Redness)	14.20 ± 1.1	15.60 ± 1.3	13.80 ± 0.9	16.00 ± 1.4	0.035	<0.001	0.080
b* (Yellowness)	6.10 ± 0.8	5.85 ± 0.7	6.30 ± 0.9	5.65 ± 0.6	0.420	0.048	0.520

^1^ Values are expressed as mean ± SD (*n* = 15 per group). Abbreviations: OL, diet supplemented with *Olea europaea* L. extract; LL, *longissimus lumborum*; PM, *psoas major*. Data were analyzed using a two-way ANOVA model. D: main effect of dietary treatment; M: main effect of muscle type; D × M: interaction between dietary treatment and muscle type. The interaction between diet and muscle (D × M) was not significant for any of the parameters (*p* > 0.05).

**Table 8:** Meat chemical composition meat (g/100 g wet weight) ^1^.

	Dietary Treatment		*p*-Value
	Control Group	OL Group	
Moisture	71.65 ± 1.93	72.00 ± 1.98	0.301
Crude protein	23.67 ± 2.42	23.58 ± 2.76	0.139
Ether extract	3.05 ± 0.63	3.03 ± 0.59	0.175
Ash	1.53 ± 0.33	1.51 ± 0.47	0.151

^1^ Values are expressed as mean ± SD (*n* = 15 per group).

**Table 9:** Physicochemical and mechanical properties of the *longissimus lumborum (LL)* and *psoas major* (PM) muscles in pigs ^1^.

Items	Diet (D)		Muscle (M)		*p*-Value		
	Control (Mean ± SD)	OL (Mean ± SD)	LL (Mean ± SD)	PM (Mean ± SD)	D	M	D × M
Cooking loss (%)	22.68 ± 1.57	16.99 ± 1.47	19.95 ± 3.02	19.72 ± 2.67	<0.001	0.850	0.910
Initial force (N/cm^2^)	5.45 ± 0.69	8.38 ± 1.00	5.21 ± 0.68	8.62 ± 1.00	<0.001	<0.001	0.120
WBSF (N)	36.60 ± 2.27	43.00 ± 3.57	38.71 ± 2.62	40.89 ± 3.22	0.002	0.045	0.750
Peak force (N)	38.35 ± 4.55	45.49 ± 3.59	38.83 ± 4.36	45.02 ± 3.79	0.005	0.015	0.820
Work (N × mm)	164.69 ± 19.5	184.99 ± 24.8	158.27 ± 19.7	191.42 ± 24.6	0.041	<0.001	0.950

^1^ Values are mean ± SD (*n* = 15 per group). Statistical significance was determined using a two-way ANOVA considering diet and muscle as fixed factors. D: main effect of dietary treatment (Control vs. OL); M: main effect of muscle type (LL vs. PM); D × M: interaction between diet and muscle. Initial force: resistance to initial compression; WBSF: Warner–Bratzler shear force; Peak force: maximum force during shear test; Work: total energy required for shearing.

**Table 10:** Fatty acid profile of *longissimus lumborum* and *psoas major* pig muscles (% of total fatty acid) ^1^.

Fatty Acid (%)	Items	Diet (D)		Muscle (M)		*p*-Value		
		Control	OL	LL	PM	D	M	D × M
Myristic	C14:0	1.85 ± 0.15	1.65 ±0.10	1.70 ± 0.12	1.80 ± 0.14	0.042	ns	ns
Palmitic	C16:0	26.80 ± 0.80	23.80 ± 0.75	25.10 ± 0.85	25.50 ± 0.70	<0.001	ns	ns
Stearic	C18:0	10.20 ± 1.28	8.13 ± 1.10	9.00 ± 1.05	9.33 ± 1.20	<0.001	ns	ns
Lignoceric	C24:0	0.02 ± 0.01	0.42 ± 0.05	0.20 ± 0.03	0.24 ± 0.04	<0.001	ns	ns
SFA (Total)		38.87 ± 3.44	34.00 ± 2.50	36.00 ± 2.80	36.87 ± 3.10	<0.001	ns	ns
Trans-Palmitoleic	C16:1 n7t	0.02 ± 0.01	0.05 ± 0.01	0.03 ± 0.01	0.04 ± 0.01	ns	ns	ns
Palmitoleic	C16:1 n7	3.00 ± 0.46	2.53 ± 0.40	2.70 ± 0.35	2.83 ± 0.42	ns	ns	ns
Trans-Oleic	C18:1 n9t	0.01 ± 0.01	0.02 ± 0.01	0.01 ± 0.01	0.02 ± 0.01	ns	ns	ns
Oleic	C18:1 n9	48.00 ± 3.14	51.35 ± 3.20	49.50 ± 2.90	49.85 ± 3.50	0.025	ns	ns
Eicosenoic	C20:1 n9	0.01 ± 0.01	0.05 ± 0.02	0.03 ± 0.01	0.03 ± 0.01	ns	ns	ns
Nervonic	C24:1 n9	0.10 ± 0.02	1.00 ± 0.15	0.50 ± 0.10	0.60 ± 0.12	<0.001	ns	ns
MUFA (Total)		51.14 ± 4.02	55.00 ± 3.80	52.77 ± 3.40	53.37 ± 3.90	0.030	ns	ns
Trans-Linoleic	C18:2 n6t	0.01 ± 0.01	0.02 ± 0.01	0.01 ± 0.01	0.02 ± 0.01	ns	ns	ns
Linoleic	C18:2 n6	7.50 ± 1.04	8.50 ± 0.90	7.90 ± 0.85	8.10 ± 0.95	0.040	ns	ns
Gamma-Linolenic	C18:3 n6	0.05 ± 0.01	0.04 ± 0.01	0.04 ± 0.01	0.05 ± 0.01	ns	ns	ns
Alpha-Linolenic	C18:3 n3	1.00 ± 0.05	1.80 ± 0.15	1.35 ± 0.10	1.45 ± 0.12	<0.001	ns	ns
Eicosadienoic	C20:2 n6	0.82 ± 0.09	0.30 ± 0.07	0.55 ± 0.06	0.57 ± 0.08	<0.001	ns	ns
Dihomo-γ-Linolenic	C20:3 n6	0.13 ± 0.04	0.11 ± 0.03	0.12 ± 0.02	0.12 ± 0.03	ns	ns	ns
Arachidonic	C20:4 n6	0.50 ± 0.06	0.43 ± 0.07	0.45 ± 0.05	0.48 ± 0.08	ns	ns	ns
EPA	C20:5 n3	0.05 ± 0.01	0.17 ± 0.03	0.10 ± 0.02	0.12 ± 0.03	<0.001	ns	ns
Docosatetraenoic	C22:4 n6	0.10 ± 0.02	0.05 ± 0.01	0.07 ± 0.02	0.08 ± 0.01	ns	ns	ns
Docosapentaenoic	C22:5 n3	0.03 ± 0.01	0.06 ± 0.01	0.04 ± 0.01	0.05 ± 0.01	<0.05	ns	ns
DHA	C22:6 n3	0.00 ± 0.00	0.01 ± 0.00	0.01 ± 0.00	0.01 ± 0.00	ns	ns	ns
PUFA (Total)		10.20 ± 0.50	11.51 ± 0.60	10.65 ± 0.55	11.06 ± 0.65	<0.001	ns	ns
n-6 Total		9.11 ± 0.74	9.45 ± 0.80	9.14 ± 0.75	9.42 ± 0.82	ns	ns	ns
n-3 Total		1.08 ± 0.18	2.04 ± 0.23	1.50 ± 0.20	1.62 ± 0.22	<0.001	ns	ns
n-6/n-3 ratio		8.43 ± 0.29	4.63 ± 0.14	6.09 ± 0.25	5.81 ± 0.20	<0.001	ns	ns

^1^ Results are expressed as % of total fatty acids ± SD. Abbreviations: MUFA, monounsaturated fatty acid; SFA, saturated fatty acid; PUFA, polyunsaturated fatty acid; n-6, omega-6; n-3, omega-3; ns, not significant. Two-way ANOVA in the *longissimus lumborum (LL)* and *psoas major* (PM) muscles in Control and OL diet.

**Table 11:** Antioxidant and oxidative stress parameters of LL and PM pig muscles under Control and OL diet.

Parameters	LL Muscle		PM Muscle	
	Control Diet	OL Diet	Control Diet	OL Diet
Total Antioxidant Activity (%)	11.05 ± 0.04 ^a^	16.70 ± 0.06 ^b^	13.01 ± 0.05 ^a^	19.84 ± 0.10 ^b^
SOD (U/mg protein) ^1^	10.98 ± 0.03 ^a^	23.78 ± 0.23 ^b^	9.87 ± 0.02 ^a^	22.31 ± 0.25 ^b^
CAT (U/mg protein) ^2^	0.42± 0.03 ^a^	0.49 ± 0.02 ^b^	0.55 ± 0.06 ^a^	0.65 ± 0.07 ^b^
MDA (nmol/mg) ^3^	0.90 ± 0.02 ^a^	0.22 ± 0.01 ^b^	0.53 ± 0.01 ^a^	0.20 ± 0.01 ^b^

^1^ Superoxide dismutase. ^2^ Catalase. ^3^ Malondialdehyde. Values are mean ± SD (*n* = 15 per group). Different letters (a, b) indicate significant differences between diets within the same muscle (*p* < 0.001).

## Data Availability

All the data are available in the manuscript.

## Supplementary Materials

The following supporting information can be downloaded at: https://www.mdpi.com/article/10.3390/ani16071009/s1, Figure S1: Chromatographic profiles of *Caiazzana* olive leaves extracts; Table S1: Phenolic acid contents (mg/g of dry extract) of *Caiazzana* olive leaves with retention time and r2.

## Abbreviations

The following abbreviations are used in this manuscript:

PM	*Psoas major*
LL	*Lo**ngissimus lumborum*
ACTH	AdrenoCorticoTropic Hormone
HPA	Hypothalamic–pituitary–adrenal
DPPH	2,2-Diphenyl-1-picrylhydrazyl
MDA	Malondialdehyde
SOD	Superoxide dismutase
CAT	Catalase
SEM-EDS	Scanning Electron Microscopy-Energy Dispersive Spectrometry
