# Supplementary material for: An Integrated Approach to Evaluate the Influence of Dietary Olea europaea L. Polyphenols on Physiological Stress, Intestinal Morphofunctional Traits, and Meat Quality in Neroametà Pigs: A Preliminary Study
Source: Animals (Basel). 2026 Mar 25;16(7):1009. doi: 10.3390/ani16071009 (PMC13072085; doi:10.3390/ani16071009)
Supplement: Supplementary file 1 [file animals-16-01009-s001.zip › animals-4212962-supplementary.pdf]

Supplementary Materials

An integrated approach to evaluate the influence of dietary *Olea europaea* L. polyphenols on physiological stress, intestinal morphofunctional traits, and meat quality in Neroametà pigs: a preliminary study

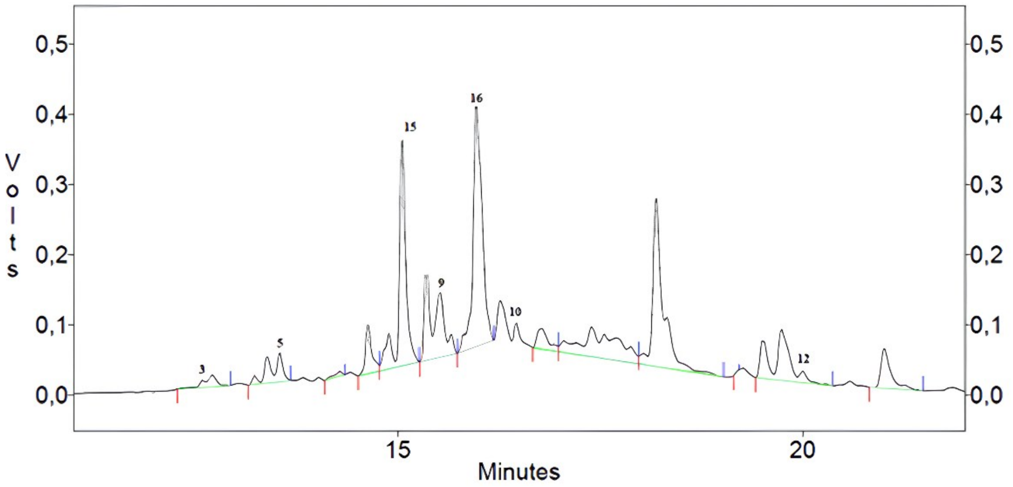

Figure S1. Chromatographic profiles of *Caiazzana* olive leaves extracts.

Table S1. Phenolic acid contents (mg/g of dry extract) of *Caiazzana* olive leaves with retention time and r^2. [1]

No. Peak	Compound	λ (nm)	RT (min)	r^2	Phenolic compounds Concentration (mg/g)
3	Tyrosol	280	13.2	0.98	0.052 ± 0.02
5	Hydroxytyrosol	280	14.2	0.97	0.067 ± 0.04
9	Luteolin-7-O-glucoside	280	15.8	0.98	0.086 ± 0.04
10	Chlorogenic acid	280	16.3	0.98	0.080 ± 0.03
12	Quercetin	280	20	0.99	0.089 ± 0.04
15	Verbascoside	280	15	0.99	0.100 ± 0.05
16	Oleuropein diglucoside	280	16	0.99	0.125 ± 0.05

[1] Values are mean ± SD (n = 3).
